# Differential Phasing between Circadian Clocks in the Brain and Peripheral Organs in Humans

**DOI:** 10.1177/0748730416668049

**Published:** 2016-10-04

**Authors:** Jacob J. Hughey, Atul J. Butte

**Affiliations:** * Department of Biomedical Informatics, Vanderbilt University School of Medicine, Nashville, Tennessee; † Institute for Computational Health Sciences, University of California, San Francisco, California

**Keywords:** circadian, gene expression, meta-analysis, mammals, phasing

## Abstract

The daily timing of mammalian physiology is coordinated by circadian clocks throughout the body. Although measurements of clock gene expression indicate that these clocks in mice are normally in phase with each other, the situation in humans remains unclear. We used publicly available data from five studies, comprising over 1000 samples, to compare the phasing of circadian gene expression in human brain and human blood. Surprisingly, after controlling for age, clock gene expression in brain was phase-delayed by ~8.5 h relative to that of blood. We then examined clock gene expression in two additional human organs and in organs from nine other mammalian species, as well as in the suprachiasmatic nucleus (SCN). In most tissues outside the SCN, the expression of clock gene orthologs showed a phase difference of ~12 h between diurnal and nocturnal species. The exception to this pattern was human brain, whose phasing resembled that of the SCN. Our results highlight the value of a multi-tissue, multi-species meta-analysis, and have implications for our understanding of the human circadian system.

In evolutionary response to the 24-h period of Earth’s rotation, diverse species—from cyanobacteria to humans—possess a molecular oscillator termed the circadian clock. In mammals, the circadian clock is involved in the daily rhythms of numerous aspects of physiology, including sleep (Borbély and Achermann, 1999), metabolism (Bugge et al., 2012), and DNA repair (Kang et al., 2009). The clock also appears to be disrupted in multiple conditions, including cancer (Cadenas et al., 2014) and major depressive disorder (Li et al., 2013).

Because the mammalian circadian clock is cell-autonomous, each organism possesses not one clock, but many (Yamazaki et al., 2000; Nagoshi et al., 2004). The “master” clock in the SCN receives information about the light-dark cycle from specialized cells in the retina (Do and Yau, 2010; Evans et al., 2011). Clocks in peripheral organs receive signals from the SCN and can respond to non-photic environmental cues, especially the feeding schedule (Mohawk et al., 2012).

Increasing evidence suggests that the alignment of clocks throughout the body is important for circadian function. In mice, the clocks in (in terms of expression of core clock genes) various organs are normally in phase with each other (Zhang et al., 2014; Evans et al., 2015). This alignment can be disrupted—i.e., peripheral clocks can be decoupled from the clock in the SCN—by a number of environmental perturbations, including cold and hunger (van der Vinne et al., 2014), or by restricting feeding to the daytime (Damiola et al., 2000); the latter has recently been shown to impair hippocampal-dependent learning and memory (Loh et al., 2015). Unfortunately, what constitutes normal alignment of circadian clocks in human organs is unknown.

Given that the core clock genes in mammals are highly conserved, one might expect that the interactions between clock genes and proteins—and therefore the clock’s dynamics—are also conserved. Indeed, mouse and human cells show similar relative phasing of clock gene expression in vitro (Hughes et al., 2009). In vivo, one might hypothesize that peripheral clocks in nocturnal and diurnal mammals would be out of phase by 12 h. Previous work, however, found that the clocks in mouse brain (nocturnal) and human brain (diurnal) were out of phase by only 6.5 h (Li et al., 2013). The reason for this discrepancy remains unclear.

Here we sought to take advantage of recently published gene expression datasets to compare the phases of circadian clocks across multiple organs in humans and across mammalian species.

## Materials and Methods

All data and code to reproduce this study are available at http://dx.doi.org/10.5061/dryad.g928q.

### Selecting the Samples

Overall, we analyzed 15 datasets of genome-wide gene expression data (5 from humans, 7 from mice, and 3 from rats) and included published results from an additional 13 studies on various diurnal and nocturnal mammals. For the datasets from human blood, we included samples corresponding to control conditions (e.g., we excluded samples obtained during sleep deprivation). For GSE45642, we only included samples from control subjects (i.e., we excluded subjects with major depressive disorder). For studies from non-human species, we included samples from wild-type animals in control conditions, including both DD (continuous darkness) and LD (alternating light-dark) regimens. See Suppl. Table S1 for the details of all datasets.

### Processing the Metadata

Studies from non-human species and studies from human brain provided the circadian time for each sample (where CT0 represents sunrise). Studies from other human organs provided the time of day for each sample (e.g., 0800 h). For the latter, we converted the time of day to circadian time using the dates provided by the authors or the average sunrise time in the respective geographic location. We validated the averaging procedure using GSE56931 (the only study for which we could make the comparison): the average sunrise time and the actual sunrise time gave nearly identical results.

The studies from human brain and one study from blood (GSE56931) provided the biological sex of the corresponding subject for each sample. For all three studies of human blood (GSE39445, GSE48113, and GSE56931), we inferred biological sex using the median expression of RPS4Y1 (prior to batch correction) across all samples for each subject, which is unambiguously low or high in females or males, respectively. For GSE56931, the inferred sex was exactly concordant with the information provided.

For the analysis of the effect of age on circadian phasing in human brain, the threshold of 40 years was chosen for consistency with prior work (Chen et al., 2016) and to ensure a similar age distribution for blood and young brain samples and a sufficient number of samples for young brain. The age distributions of the younger and older groups were 29.7 ± 8.9 years and 58 ± 10 years, respectively (M ± SD).

### Processing the Expression Data

Gene expression from microarray data was processed using metapredict, which maps probes to Entrez Gene IDs and performs intra-study normalization and log-transformation (Hughey and Butte, 2015) (https://github.com/jakejh/metapredict). For GSE72095, which is an RNA-seq dataset, we downloaded the sequencing reads from the NCBI Sequence Read Archive (SRA), then used kallisto to quantify transcript abundance in units of transcripts per million (tpm) (Bray et al., 2016). To make the RNA-seq data comparable with the microarray data, we first used the mapping of Ensembl Transcript IDs to Entrez Gene IDs to calculate “gene” abundances as the sum of tpm values for transcripts mapping to a given gene, then used the formula log(tpm+1) in the analysis. In each dataset, genes from non-human species were mapped to their respective human orthologs using information from NCBI (Fong et al., 2013).

To reduce the effects of inter-individual and inter-organ variability in gene expression in humans, we used ComBat to adjust the expression values within each human dataset (Johnson et al., 2007). Within each dataset from human blood, in which there were 7-14 samples taken throughout the day for each subject, we applied the batch correction by subject. Within each dataset from human brain, in which all samples from a given subject corresponded to the same circadian time, we applied the batch correction by anatomical area (correcting by subject would remove all circadian variation). For the results in Figure 2B-C and Figures 3-4, we also applied a batch correction to adjust for expression differences between the two datasets from human brain.

### Analyzing Circadian Gene Expression

Within each dataset, we used ZeitZeiger to fit a periodic smoothing spline, $f_{j}\left(t\right)$, to the expression of each gene, j, as a function of circadian time, t (Hughey et al., 2016; Helwig and Ma, 2014) (https://github.com/jakejh/zeitzeiger). To constrain their flexibility and prevent overfitting, all spline fits were based on three knots.

We estimated the time of peak expression for gene, j, as $argmaxf_{j}\left(t\right)$, and the time of trough expression as $argminf_{j}\left(t\right)$. Differences between peak times or trough times were calculated using ZeitZeiger, and accounted for the periodic nature of circadian time (e.g., CT2 is 6 h ahead of CT20).

We calculated the signal-to-noise ratio of circadian rhythmicity as $SNR_{j}=\frac{max\left(f_{j}\left(t\right)\right)-min\left(f_{j}\left(t\right)\right)}{s_{j}}$, where $s_{j}$is the root-mean-square error of the periodic spline fit.

The mean peak times in Figure 3 were calculated as circular means; i.e.,

$$\bar{t}=\frac{24}{2\pi }atan2\left(\sum_{i=1}^{n}sin\left(t_{i}\frac{2\pi }{24}\right),\sum_{i=1}^{n}cos\left(t_{i}\frac{2\pi }{24}\right)\right).$$

Simulations to determine accuracy of peak and trough time detection assumed sinusoidal periodicity (period of 24 h), i.i.d. additive Gaussian noise, and that observations were randomly spaced in time. For each combination of number of observations and expected signal-to-noise ratio, we generated 100 simulations. For each simulation, we fit a periodic smoothing spline (based on three knots) and estimated peak and trough times in the same way as for the actual gene expression data.

## Results

### Clock Gene Expression in Human Blood and Human Brain

We first assembled five publicly available datasets of genome-wide circadian gene expression in humans: three from blood (Archer et al., 2014; Möller-Levet et al., 2013; Arnardottir et al., 2014) and two from brain (Li et al., 2013; Chen et al., 2016) (Table 1 and Suppl. Table S1). Each dataset from blood consisted of ~8 samples taken throughout the day for each participant (~20 participants per study). Both datasets from brain were based on postmortem tissue from multiple anatomical areas: amygdala, anterior cingulate cortex, cerebellum, dorsolateral prefrontal cortex, hippocampus, and nucleus accumbens (GSE45642); Brodmann’s areas 11 and 47 of the prefrontal cortex (GSE71620). Circadian time for each sample from the brain was based on the respective donor’s time and date of death and geographic location (55 donors for GSE45642, 146 donors for GSE71620)

**Table 1.** Datasets of circadian gene expression in humans.

Dataset	Reference	Organ	Condition	Subjects	Samples
GSE39445	Möller-Levet et al., 2013	blood	living	24	221
GSE48113	Archer et al., 2014	blood	living	22	147
GSE56931	Arnardottir et al., 2014	blood	living	14	130
GSE45642	Li et al., 2013	brain^a^	postmortem	55	269
GSE71620	Chen et al., 2016	brain^b^	postmortem	146	292

a. Amygdala, anterior cingulate cortex, cerebellum, dorsolateral prefrontal cortex, hippocampus, and nucleus accumbens. b. Brodmann’s areas 11 and 47 of prefrontal cortex. See Suppl. Table S1 for more details.

Because most circadian gene expression is organ-specific (Zhang et al., 2014), we limited our analysis to genes known to be part of the core circadian clock (or a direct output of the clock, in the case of *DBP*). In mice, these are among the few genes whose expression shows circadian oscillations at the same phase across organs. Expression of the core clock genes is an accurate indicator of internal circadian time and can be used to detect when the clock is phase-shifted (Hughey et al., 2016). For each clock gene in each dataset, we quantified the signal-to-noise ratio (SNR) of circadian rhythmicity of expression (Suppl. Fig. S1 and Materials and Methods). Clear rhythmicity (SNR > 1) in both blood and brain was exhibited by seven clock genes (*ARNTL, DBP, NR1D1, NR1D2, PER1, PER2*, and *PER3*), and we focused on these for the remainder of the study.

**Figure 1.**
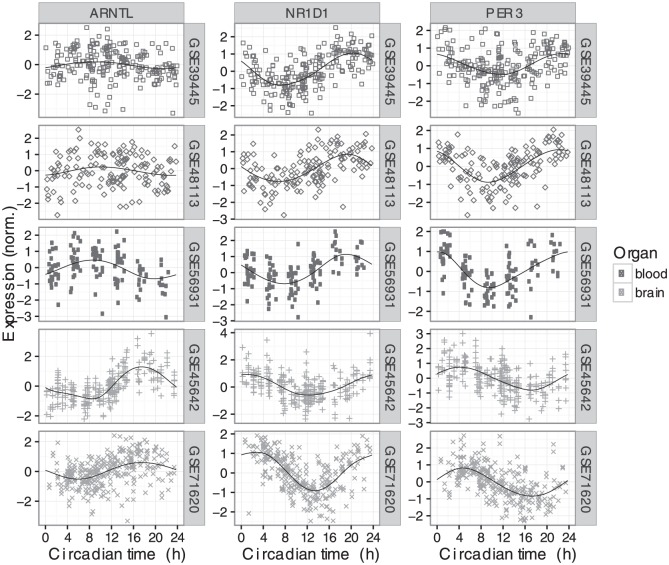
Circadian expression of three clock genes across five datasets in two human organs (top three rows are blood, bottom two rows are brain). Circadian time 0 corresponds to sunrise. Each point is a sample and the shape indicates the dataset. In each plot, the black line shows the periodic smoothing spline corresponding to the mean expression over time. Expression values for each gene in each dataset were scaled to have mean of zero and an SD of 1. Expression of the four other clock genes considered in this study is shown in Suppl. Fig. S2.

**Figure 2.**
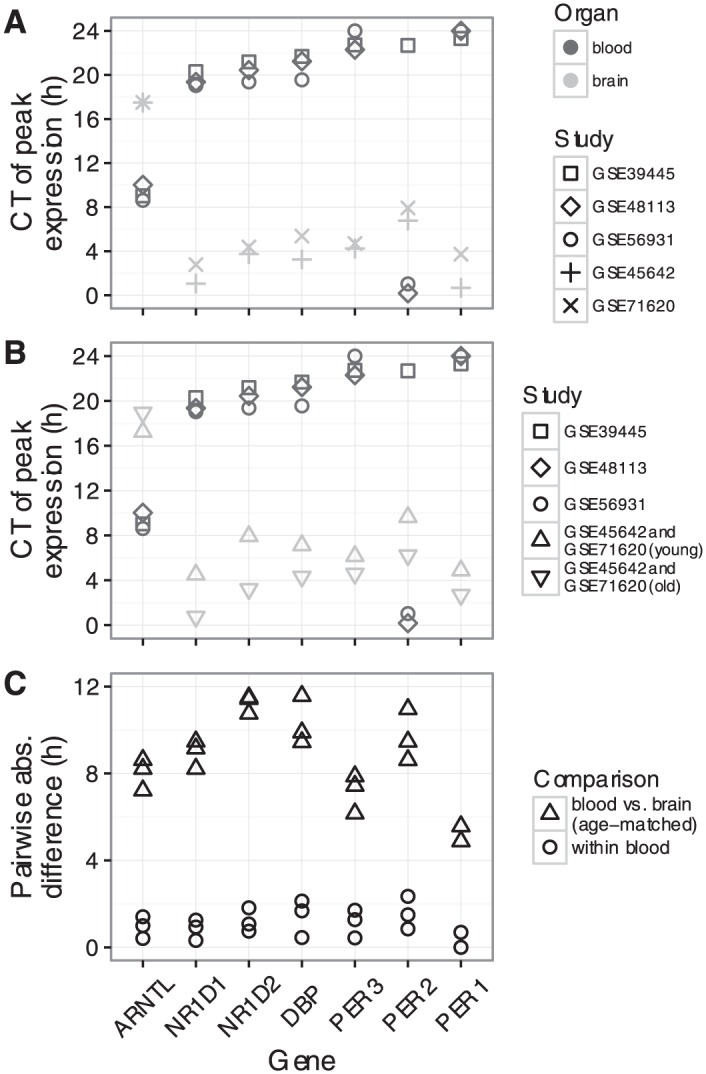
Quantifying the phase difference between human blood and human brain. **(A)** Time of peak expression for each clock gene in each dataset. Symbols as in Figure 1. CT0 is equivalent to CT24. **(B)** Similar to A, except that samples from the two brain datasets have been merged, then split by subject age (≤40 or >40 years). **(C)** Pairwise absolute differences in peak time within and across organs. Each point corresponds to one pairwise comparison. Circles correspond to comparisons between the three blood datasets. Upward-pointing triangles correspond to comparisons between each blood dataset and the merged, young brain dataset (upward-pointing triangles in **B**).

We next examined the expression of those seven genes in each dataset as a function of circadian time (CT0 corresponds to sunrise; Fig. 1 and Suppl. Fig. S2). For each gene, the circadian phase of expression was similar across the datasets from the same organ (blood or brain). Surprisingly, however, when comparing clock gene expression between organs, we observed a consistent phase difference. To quantify this phase difference, we estimated the time of peak expression for each gene in each dataset (Fig. 2A and Materials and Methods). In simulations, given data with 200 samples and SNR = 2 (typical for human data in this study), our method estimated peak time with a 95% confidence interval of 1.14 h (Suppl. Fig. S3). For six of the seven genes, peak time in brain was 6-8 h later (equivalently, 16-18 h earlier) than peak time in blood. For example, the expression of *NR1D1* peaked near CT20 in blood, but near CT2 in brain.

We then confirmed that the phase difference was not due to differences in the sex ratio or age distribution between the datasets. Although the sex ratio differed across datasets for the two organs (~50% female for blood and ~20% female for brain), we observed no consistent difference in peak times between males and females within either organ (Suppl. Fig. S4A). More significantly, the participants in the blood datasets tended to be much younger (27 ± 5 years) than the donors in the brain datasets (52 ± 15 years). Therefore, we merged the two brain datasets, split the samples into two groups based on the donor’s age (≤40 or >40 years), and calculated the peak times separately for each group. Consistent with previous work (Chen et al., 2016), clock gene expression was phase-advanced in samples from older compared with younger individuals (Fig. 2B). Consequently, after controlling for age, the absolute phase difference between blood and brain across all seven genes was 8.6 ± 2.1 h (M ± SD; median 8.6 h; Fig. 2C). We obtained similar results when quantifying the absolute phase difference using the trough time instead of the peak time (8.3 ± 1.8 h and median 8 h; Suppl. Fig. S5). In addition, when we calculated the peak time for each gene relative to the peak time of *ARNTL*, the differences between the two organs largely disappeared (Suppl. Fig. S4B; the choice of reference gene is arbitrary and does not affect the results). Taken together, these results suggest that the circadian clocks in human brain and human blood are progressing similarly, but are phase-shifted from one another.

Because the datasets from brain were based on tissues from postmortem donors, we considered that the observed circadian phasing might be an artifact of the clock continuing to progress after the official time of death. Based on the postmortem interval for samples from GSE71620, however, even 8 h of postmortem progression of the clock could not explain the phase difference between blood and brain (Suppl. Fig. S6 and S7A). Because the postmortem interval was strongly dependent on the time of death (Suppl. Fig. S7B-C), longer-lasting postmortem progression would imply that the variation in gene expression between samples is caused not by variation in circadian time of death, but by some other factor. Given that many of the top oscillatory genes in both brain datasets (assuming no postmortem progression) are known to be part of or regulated by the circadian clock (Li et al., 2013; Chen et al., 2016), this seems unlikely.

### Clock Gene Expression across Mammalian Species and Organs

We next expanded our analysis to include data from two additional human organs and from three other mammalian species: mouse, rat, and Siberian hamster (Suppl. Table S1). Where possible for a given species and organ, we included multiple independent datasets from multiple light-dark regimens (predominantly LD 12:12 and DD). For human skin, human hair follicle, and Siberian hamster heart, we curated the times of peak expression from published results (Spörl et al., 2012; Akashi et al., 2010; Crawford et al., 2007). For all other datasets (Bray et al., 2008; Dyar et al., 2014; Haspel et al., 2014; Hughes et al., 2009; Zhang et al., 2014; Boorman et al., 2005; Almon et al., 2008a; Almon et al., 2008b), we analyzed the publicly available microarray data identically to how we analyzed the data from human blood and brain (Suppl. Fig. S8). Clock genes in non-human species were then mapped to the respective human orthologs.

**Figure 3.**
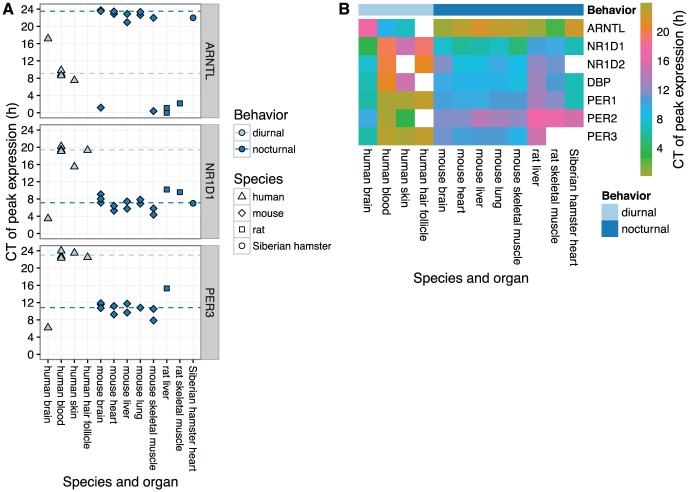
Circadian phasing of clock genes across mammalian species and organs. **(A)** Time of peak expression for three sets of clock gene orthologs. Each point represents the peak time for one gene in one dataset and organ. Dashed lines show the circular mean peak time for diurnal (excluding human brain) and nocturnal species, calculated as described in the Materials and Methods. For human brain, samples from subjects older than 40 years were excluded and the two datasets were merged as in Figure 2B-C. Plots of peak times for the other clock genes considered in this study are shown in Suppl. Fig. S9. The three points for mouse brain were measured in brain stem, cerebellum, and hypothalamus (all from GSE54650). **(B)** Heatmap of peak time for seven clock genes across species and organs. In the case of multiple datasets per combination of gene and species-organ, the color is based on the circular mean peak time. White squares correspond to genes that either were not measured or did not show rhythmicity in the respective species-organ.

In all organs from nocturnal species, each of the seven clock genes showed similar circadian phasing (Fig. 3 and Suppl. Fig. S9). In particular, expression of the *ARNTL* ortholog peaked near CT0 (equivalent to CT24), and expression of the other genes peaked between CT8 and CT14. Interestingly, peak times in rat tended to be slightly later than those in mouse and Siberian hamster.

Compared to peak times in nocturnal species, peak times in almost all organs from humans were shifted by ~12 h (Fig. 3 and Suppl. Fig. S9). The exception was human brain, whose phase signature was distinct from human blood, skin, and hair follicle. Again, peak times relative to *ARNTL* were similar for organs in all species, both nocturnal and diurnal (Suppl. Fig. S10).

### Clock Gene Expression in the Mammalian Suprachiasmatic Nucleus

Finally, we expanded our analysis to include studies of clock gene expression in the site of the master clock, the SCN (Albrecht et al., 1997; Maywood et al., 1999; Miyake et al., 2000; Yan et al., 1999; Vosko et al., 2009; Lambert, 2005; Caldelas et al., 2003; Mrosovsky et al., 2001; Pembroke et al., 2015; Takumi et al., 1998b; Takumi et al., 1998b) (Suppl. Table S1). Most of these studies, which were conducted on various diurnal and nocturnal rodents, measured the expression of the respective *PER1* and *PER2* orthologs by in situ hybridization. From these studies, we curated peak times from the published result. The two datasets of genome-wide gene expression in the mouse SCN, on the other hand, were analyzed identically to that of human blood and brain.

**Figure 4.**
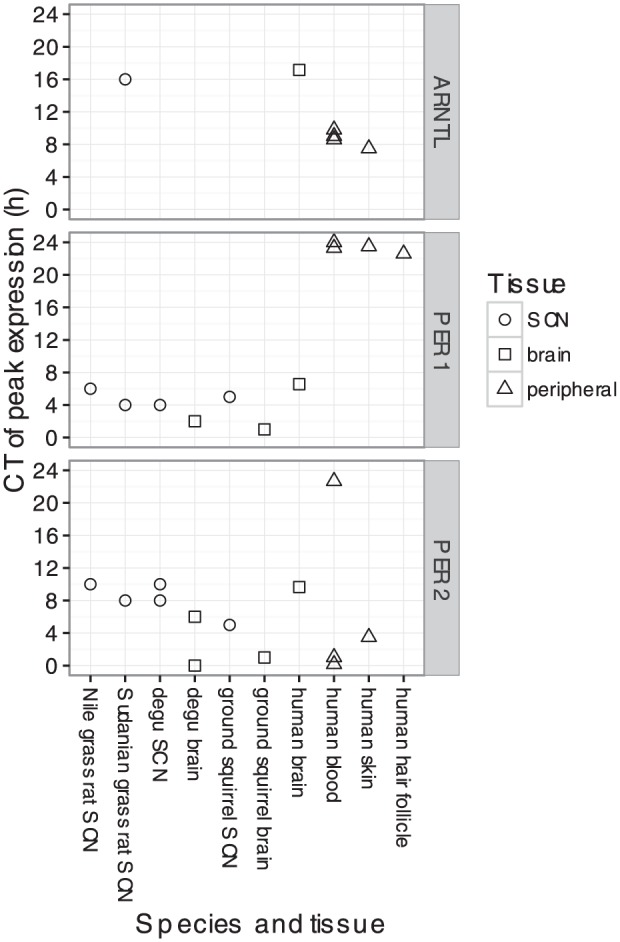
Circadian phasing of clock gene expression in various tissues (including the SCN) of various diurnal mammalian species. Data from diurnal rodents was curated from published studies that measured transcript levels using in situ hybridization.

In contrast to tissues outside the SCN, where peak times differed by 12 h between diurnal and nocturnal mammals, peak times in the SCN were nearly identical across species (CT5 for *PER1* and CT9.5 for *PER2*; Suppl. Fig. S11). As a result, peak times in the SCN were 4-8 h later than that of most other organs of diurnal mammals (Fig. 4) and 4-8 earlier than that of other organs of nocturnal mammals (Suppl. Fig. S12). Interestingly, the phasing of clock gene expression in human brain was similar to that of the SCN (Fig. 4).

## Discussion

Here we described the most comprehensive analysis to date of the circadian phasing of clock gene expression in mammals. Our results indicate conservation of not only the clock’s molecular components, but also its dynamics (in terms of relative phasing of gene expression). What differs between species is not the clock itself but the alignment of the clocks in various organs with each other and with the external environment. The clock in the SCN, consistent with its responsiveness to the light-dark cycle, showed the same circadian phasing in diurnal and nocturnal species. Clocks outside the SCN, in general, were phase-shifted by ~12 h between diurnal and nocturnal species. The exception to this pattern was human brain, whose clock gene expression was distinct from that of other peripheral organs in humans and other diurnal mammals and resembled that of the mammalian SCN.

In this study, we relied on datasets in which the time of day for each sample was known. Although many genes show circadian rhythmicity in expression (up to 50% in mice) (Zhang et al., 2014), such datasets are unfortunately in the minority. As a result, circadian variation could be a confounding factor in many analyses of genome-wide gene expression. In the future, it may be possible to detect, correct for, and learn from unannotated circadian variation in publicly available data, perhaps using methods developed primarily for the cell cycle (Buettner et al., 2015; Leng et al., 2015).

In mice, circadian gene expression can vary from one brain region to another (Feillet et al., 2008; Zhang et al., 2014). Because previous analyses of human brain revealed similar patterns of circadian gene expression in each region (Li et al., 2013; Chen et al., 2016), our analysis combined the data from the various regions of human brain in each dataset. Nonetheless, there are many regions of the human brain for which circadian gene expression has not yet been measured, most notably the SCN itself. Moreover, circadian rhythms in different cell types of the nervous system (e.g., neurons and glia) have yet to be thoroughly examined.

Although the two datasets from human brain were the only ones based on tissue from postmortem donors, our analysis suggests that the observed circadian phasing is unlikely to be an artifact of the tissue collection procedure or the postmortem interval. Two additional points support this view. First, both datasets only included samples from donors who suffered rapid death, avoiding the drop in pH and alterations in gene expression associated with prolonged agonal states (Li et al., 2004). Second, the circadian phasing in the human brain is consistent across multiple clock genes and both datasets, even though the two datasets include samples from different regions of the brain and were collected by different institutions. That said, it is important that these results be replicated in additional studies.

Although the details are unclear, the SCN seems to coordinate the clocks throughout the body using a combination of neural and humoral cues (Dibner et al., 2010) and temperature (Buhr et al., 2010). The alignment of clocks in non-SCN tissues to behavior rather than to the light-dark cycle suggests that the mechanism by which the SCN communicates with other tissues differs between diurnal and nocturnal mammals. Our results also imply that the SCN in humans communicates qualitatively differently with other regions of the brain than with organs outside the brain. Due to the paucity of data from non-human diurnal mammals, it is unclear whether this differential communication is specific to humans or a general property of diurnality. One possible explanation for the former could be the modern environment, which contains many features that affect the clock, including caffeine (Burke et al., 2015), social jetlag (Wittmann et al., 2006), night-time exposure to artificial light (Gooley et al., 2011), and decreased exposure to sunlight (Wright et al., 2013). Current evidence indicates that the phase shifts induced by these conditions (~1 h) are considerably less than the phase difference we observed between brain and other organs. Even so, it seems reasonable to wonder if the circadian phase of the human brain is somehow related to the unnatural environment in which many humans live.

Recent evidence suggests that the circadian clock is an important factor in the response to many therapeutics (Zhang et al., 2014). Our observations are relevant to the future of chronotherapy for two reasons. First, they can serve as guidelines for how to convert circadian drug delivery schedules from one species to another. Second, they imply that, in humans, the optimal timing for a drug could depend on the anatomical site of action.

The adverse effects of shift work and jet lag are thought to be mediated by misalignment between the external environment and the internal circadian system (Reid and Zee, 2009). Based on our findings, we speculate that such perturbations may also cause misalignment between the clocks throughout the body. Either way, further work is needed to determine how the circadian phases of different organs drive circadian rhythms in physiology, and how the alignment between clocks is perturbed in various pathophysiological states.
